# Vector Arithmetic in the Triangular Grid

**DOI:** 10.3390/e23030373

**Published:** 2021-03-20

**Authors:** Khaled Abuhmaidan, Monther Aldwairi, Benedek Nagy

**Affiliations:** 1 Department of Computing and IT, Global College of Engineering and Technology, CPO Ruwi 112, Muscat Sultanate P.O. Box 2546, Oman; 2 College of Technological Innovation, Zayed University, 144534 Abu Dhabi, United Arab Emirates; monther.aldwairi@zu.ac.ae; 3 Department of Mathematics, Faculty of Arts and Sciences, Eastern Mediterranean University, via Mersin 10, Famagusta 99450, Turkey

**Keywords:** vector addition, nontraditional grid, triangular grid, discretized translations, digital geometry, triangular symmetry, vector arithmetic, coordinate systems, nonlinearity

## Abstract

Vector arithmetic is a base of (coordinate) geometry, physics and various other disciplines. The usual method is based on Cartesian coordinate-system which fits both to continuous plane/space and digital rectangular-grids. The triangular grid is also regular, but it is not a point lattice: it is not closed under vector-addition, which gives a challenge. The points of the triangular grid are represented by zero-sum and one-sum coordinate-triplets keeping the symmetry of the grid and reflecting the orientations of the triangles. This system is expanded to the plane using restrictions like, at least one of the coordinates is an integer and the sum of the three coordinates is in the interval [−1,1]. However, the vector arithmetic is still not straightforward; by purely adding two such vectors the result may not fulfill the above conditions. On the other hand, for various applications of digital grids, e.g., in image processing, cartography and physical simulations, one needs to do vector arithmetic. In this paper, we provide formulae that give the sum, difference and scalar product of vectors of the continuous coordinate system. Our work is essential for applications, e.g., to compute discrete rotations or interpolations of images on the triangular grid.

## 1. Introduction

**Figure 1:**
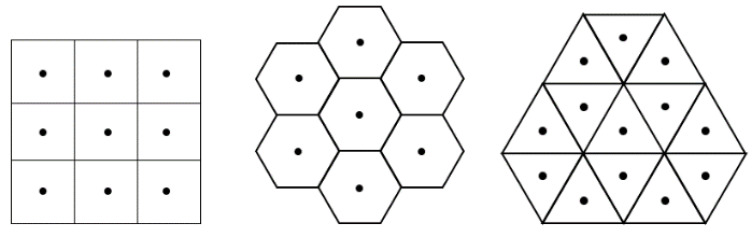
The three regular grids: the square, the hexagonal and the triangular grids and their grid points (midpoints of the pixels).

On the one hand, vector arithmetic, addition, subtraction and scalar product of vectors are base of analytic and coordinate geometry, physics and other disciplines. On the other hand, digital geometry, as a part of discrete mathematics, including, e.g., the geometry of the computer screen, is the scientific field of geometric properties of digital images. Basically, digital geometry deals with points addressed by integer coordinates in Euclidean space and it is considered to be its digitized model. However, there are fundamental differences between Euclidean and digital geometry, e.g., digital images are consisting of a finite set of pixels. While in Euclidean geometry there are infinitely many points between any two distinct points; in digital geometry, the concept of neighborhood plays a central role. Moreover, the underlying grid takes matter, since both the representations and properties of the images, and thus the possible operations on them, depend on the grid itself. The operations should work on sets of discrete points addressed with integer coordinates. There are three regular tessellations of the two-dimensional Euclidean space, i.e., the plane: The square, the hexagonal and the triangular grids are shown in Figure 1; their names come from the shape of the pixels used as tiles [1].

The Cartesian coordinate system fits very well to the square grid because it is an orthogonal coordinate system having axes parallel and orthogonal to the sides of the pixels. Also, in coordinate geometry, the Euclidean space is usually denoted by ${\mathbb{R}}^{2}$; and thus, its restriction to integer-valued points gives exactly the usual representation ${\mathbb{Z}}^{2}$ of the square grid. The two coordinates in both cases (plane and discrete grid) are independent of each other. Since these coordinate systems are well studied and widely known, hardware industry and most of the applications are using this grid. The dual of the square grid (that is, the grid formed by the nodes, which are the crossing points of the gridlines) is also a square grid; therefore, essentially, the same coordinate system is used to address either the pixels or the nodes of the grid. The usual vector arithmetic is based on Cartesian coordinate system when the Euclidean space or the rectangular grids are used, in the latter case both the nodes/vertices of the grid and the Voronoi cells (pixels or voxels) can be addressed by Cartesian vectors. Working with digital images, we may need to perform operations that do not map the grid into itself, e.g., zooming or rotations. As we have already mentioned, the Cartesian coordinate system allows using real numbers, and intermediate computations can result those, then a digitization operation can easily be defined by rounding operation to get the final result as a digital image. On the other hand, there are several studies that show problems, paradoxes of digital geometry of the square grid, e.g., the diagonals of a usual chessboard as lines cross each other without having a common pixel (this is a well-known shortcoming, a topological paradox of the square grid and there are various sophisticated techniques to avoid it) [2]. Both of the basic digital distances on the square grid, the Manhattan taxi-cab distance (same as L_1_ distance) and the chessboard distance (same as L_∞_ distance) have very large rotational dependency. The same digital distance to the axis direction and to the direction precisely between two axes (i.e., 45°) have more than 40% difference in Euclidean distance. Therefore, in many times, it is worth to consider the other two grids also in applications to avoid some of the disadvantages of the square grid and, at the same time, gaining some of the advantages of the other grids. Some of these advantages are recalled in the next part.

**Figure 2:**
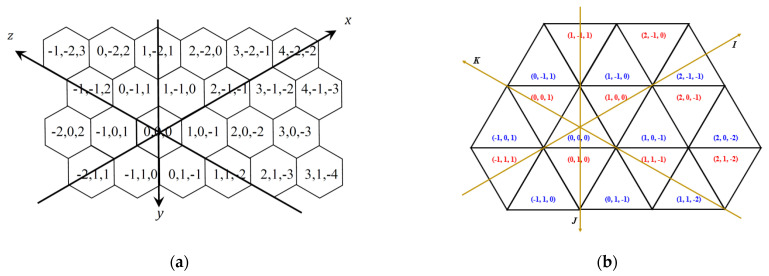
(**a**) The discrete symmetric coordinate system for the hexagonal and (**b**) for the triangular grids. The coordinate values can also be considered to be assigned the midpoints of the corresponding pixels.

The hexagonal grid, tiling the plane by same size regular hexagons (hexels or hexagonal pixels), has been used for decades in image processing applications [3], in cartography [4,5], in biological simulations [6] and in other fields, since the digital geometry of the hexagonal grid provides better results than the square grid in various cases. It is the simplest grid, in the sense that there is only one type of widely used neighborhood among the hexels, opposite to the two types of neighbors in the square grid [7]. The six neighbors of a hexel can be seen in Figure 1 in the middle. A coordinate system with zero-sum triplets can be used to describe the hexagonal grid capturing nicely the symmetry of the grid [8]. This coordinate system (see also Figure 2a) allows also real numbers to use to describe e.g., rotations that may not map the hexagonal grid to itself [9]. Moreover, a useful digitization operator is provided as well. This coordinate system appears also in [10,11] for various applications in imaging related disciplines.

In contrast to the square grid, the dual of the hexagonal grid is not the hexagonal, but the triangular grid. Thus, the triangular grid has similar symmetric properties as the hexagonal grid has. Consequently, in [12,13,14] integer coordinate triplets with sum 0 and 1 are used to represent the trixels (triangle pixels). The two different sum values reflect the two different orientations of the triangle tiles. Figure 2b shows a part of the grid with the assigned triplets. Of course, the three values are not independent for the hexagonal and triangular grids, since they are also 2D grids. To work with various algorithms in computer graphics and image processing that may not map the grid into itself, one needs the continuous extension of this coordinate system as well, which was recently developed [15]. This continuous coordinate system for triangular grid (Ω) is used to describe every point of the plane with a unique coordinate triplet. Moreover, this system can be seen as an extension of the discrete coordinate systems of the hexagonal and triangular grids. However, since the vectors of the grid fulfilling some constraints (e.g., at least one of the coordinates is an integer and the sum of the three coordinates is in the closed interval [−1,1]), the vector addition (that is closely connected to translations of images [16]) and other operations with these vectors are not straightforward. This is the topic of this paper: as a continuation of our earlier paper [15], we provide a procedure to add two (or more) vectors, to subtract vectors, and to compute the scalar product of a vector (with integer coefficient) on the continuous coordinate system for the triangular grid. Although we work with continuous coordinate system which uses real numbers, our work is essential in discrete mathematics, especially, in digital geometry to work, e.g., with digital images on the triangular grid. We should also note that hexagonal, triangular, honeycomb and other related grid structures are used in various other fields, e.g., in networks [12,13,17,18,19], in fractional calculus [20,21], in 3D printing [22], in chemical and physical modelling [23] and simulations [24,25], and in city planning [26], where continuous transformations play also crucial roles, thus our result may be applied. Additionally to the above mentioned fields, triangular grid is applied in skeletonization and thinning algorithms [27,28], in discrete tomography [29] and in cartography. The importance of the triangular grid can be underlined by the following facts. Firstly, it is the simplest in the sense that it is based on the simplest regular polygons, on triangle pixels. Then, similarly to the hexagonal grid, it has more symmetry axes than the square grid has; moreover, rotations with smaller angle (e.g., 60°) could map the grid into itself than the angle needed on the square grid (90°). However, the square and the triangle can be divided into similar, but (e.g., 4 or 9) smaller sized shapes, allowing an easy way of changing the resolution of images on these two grids; which does not go in such a simple way on the hexagonal grid. Finally, based on the three types of neighbor pixels [27], there is a wider flexibility for digital distances [14,30] to approximate better the Euclidean distance, and at the same time, to have digital distances with lower rotational dependence than on the other two regular grids. To establish the connection between continuous and discrete planes is important for engineering applications as well. The non-linearity of our system could be applied in the case of visual projections in self-driving car scenarios, such as assessing the splay angles from lateral offset and vice-versa [31] and also in the case of how humans project their memory recordings in memory coordinate spaces [32].

The structure of this paper is as follows. In the next section, as preliminaries, we recall some basic facts about transformations on the square grid and we recall the continuous coordinate system for the triangular grid (Ω) with conversions to/from the Cartesian coordinate system. The main contribution of this paper, the addition of vectors in the continuous coordinate system for the triangular grid will be investigated and described in Section 3, while other arithmetic operations and an application are given in Section 4. Concluding remarks will closes this paper in Section 5.

## 2. Preliminaries

In this section, we show, first, how vector addition is related to translations on the traditional grids, i.e., on the square grid. Then, we recall the continuous coordinate system for the triangular grid with some of its crucial properties from [15].

### 2.1. Vector Additions as Translations

Translation is an isometric transformation. Any point (*x*,*y*) of the plain can be translated by any vector (*t_x_*,*t_y_*)∈$\;{\mathbb{R}}^{2}$ resulting in the point (*x* + *t_x_*,*y* + *t_y_*)∈$\;{\mathbb{R}}^{2}$. Observe here that the coordinates of the point can also be seen as a vector from the origin to the point, and thus, the translation coincides with a vector addition which is a simple algebraic operation in this case.

Isometric transformations are well known basics of Euclidean geometry. However, more and more of our world become digital, i.e., we have digital images on our computers, smartphones, etc. It is assumed and expected that isometric transformations have a similar role in discrete/digital geometry. The digitized variants of the transformations are somewhat close to their original Euclidean, continuous variants. On the other hand, they usually do not satisfy the same properties, as usually bijectivity and/or transitivity etc. could fail. In general, these properties are hard to retain in the discrete spaces [33], whenever considering a discretized form of an arbitrary Euclidean transformation. Thus, discrete and continuous transformations yield very different theories [34]. Therefore, discrete transformations are still a hot topic of research both in theory and applications.

**Figure 3:**
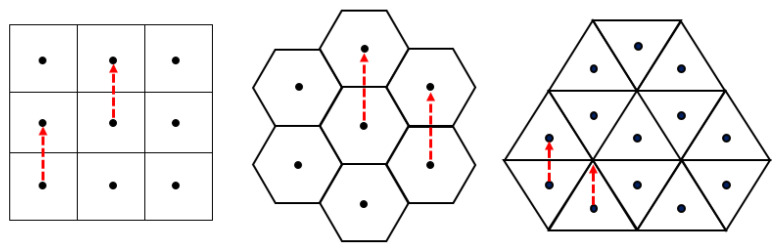
Any grid-vector of specific length and direction will lead to a grid-point in the square and the hexagonal grids but not in the triangular grid.

Translations defined on ${\mathbb{Z}}^{2}$, on the square grid, are simple and essential for various transformations in several applications related to 2D image processing such as image matching. Whenever the translation vector is also an integer vector (i.e., it belongs to ${\mathbb{Z}}^{2}$), again a simple vector addition gives the result. Further, any digital picture on the square grid can be translated by any vector of ${\mathbb{R}}^{2}$. However, if the translation vector is not in ${\mathbb{Z}}^{2}$, the result by simple vector addition will not be in the target ${\mathbb{Z}}^{2}$ anymore. Thus, a so-called digitization operation is needed to be performed after the translation to ensure the result to be again a digital image. In this way, a combination of the Euclidian translation defined on ${\mathbb{R}}^{2}$ with a digitization operator that maps the outcomes back into ${\mathbb{Z}}^{2}$ is applied. The square and the hexagonal grids are point lattices, because any of the grid-vectors is taken from any grid-point, it will always end up at a grid-point. Consequently, a similar vector-addition technique (with a fairly simple digitization operation) works also on the hexagonal grid for computing translations [9]. The digitization process plays important roles on grids when such discretized/digital operations are considered which may not be bijective [33,35], see Figure 3.

Here we are working on the triangular grid, which is, in fact, not a point lattice: it is not closed under the addition of grid-vectors. This fact gives a mathematical challenge. Those specific isometric transformations of the triangular grid are described in [36,37] that map the grid into itself; but general transformations that could map some grid-points out of the grid were not considered yet in detail. Actually, with the provided features of the continuous coordinate system, one may perform these types of transformations. Consequently, as the next step towards this, we introduce a procedure of vector addition in this system, which is considered as a basis of various types of transformations, including translations. Now, we recall the used coordinate systems for the triangular grid.

### 2.2. Coordinate Systems for the Triangular Grid

**Figure 4:**
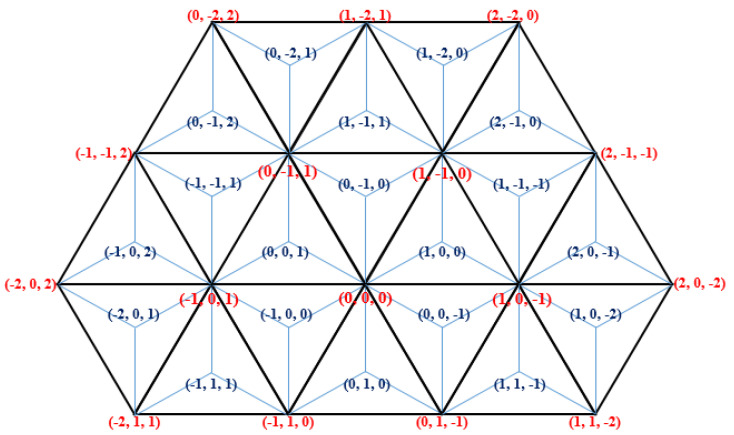
The symmetric coordinate system for the trihexagonal grid can also be used for the triangular grid and also for its dual, for the hexagonal grid, at the same time.

First, very briefly we write about a discrete coordinate system that is the base of the continuous coordinate system. In [38], various discrete coordinate systems were used for a family of various triangular grids, in each of them, integer triplets were used to address the pixels of the considered grid. These coordinate systems are symmetric coordinate systems reflecting the symmetry of the grids; the three coordinate axes have angles 120°. We start with the coordinate system for the trihexagonal grid (also called 3-planes triangular grid in [38]). The midpoints of the triangles (drawn in black color) are addressed with integer triplets with sum + 1 and −1, respectively. Notice that there are two different orientations of trixels. They are called “positive” ∆ and “negative” ∇ triangles, respectively. See Figure 4. Observe that each triplet assigned to a midpoint (see the blue triplets) builds up from the coordinate values shared by exactly two of the corners of the given trixel (see the three red triplets around each blue triplet).

**Figure 5:**
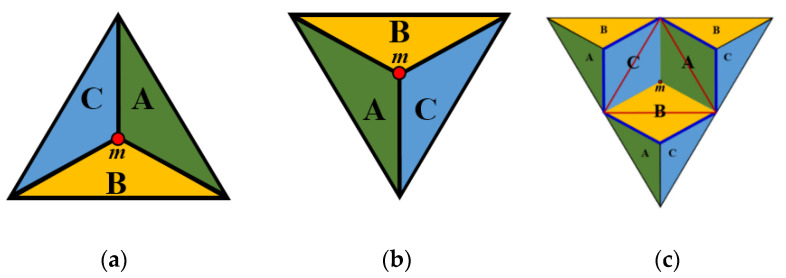
(**a**,**b**): Dividing each triangle to three areas—A, B and C. The letters assigned to the isosceles triangles are based on the orientation of sides. (**c**): The areas actually rhombuses in the plane.

Now we show the extension of the previous system to the whole plane [15]. The continuous coordinate system Ω uniquely addresses every point of the plane. It is a combination of discrete triangular coordinate systems using only integer values, including the one we have just mentioned, with the barycentric coordinate system by Möbius (see, e.g., [1,39]). Each trixel, i.e., equilateral triangle of the triangular grid (drawn by black lines in Figure 4) is divided into three inner obtuse-angled triangles, which take areas A, B, and C, as shown in Figure 5.

**Figure 6:**
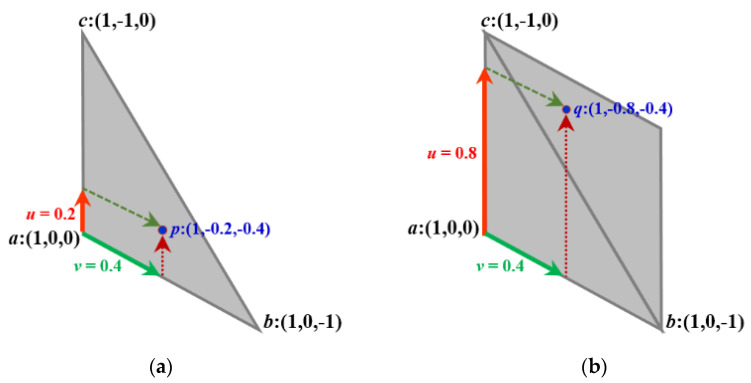
A composition of the barycentric technique and discrete coordinate system to address points *p* and *q* in the triangular plane by coordinate triplets in (**a**,**b**), respectively.

**Figure 7:**
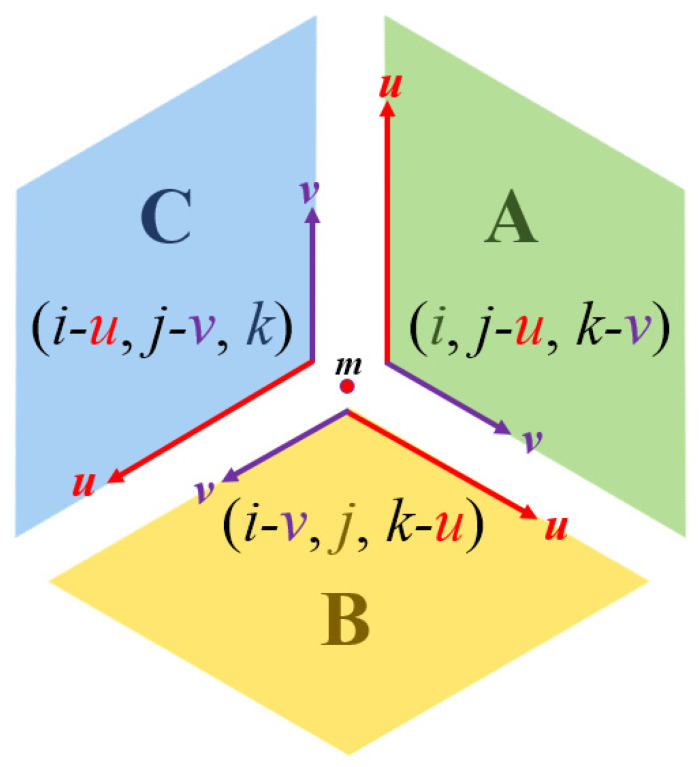
Addressing the points in a hexagon containing one area of each type, where the midpoint (*m*) of the hexagon has coordinates (*i*, *j*, *k*) and 0 *≤ u ≤* 1 and 0 *≤ v ≤* 1 in each area.

The coordinate triplets with sum + 1 and −1 are used to represent the midpoints (represented by *m* in Figure 5), depending on the orientation of the original triangle. Based on the coordinates of *m* we compute the coordinates of a point in any of the three regions (A, B, and C). Notice, that the regions in the grid, in fact, are rhombuses, thus any point inside can be addressed by two fractional values *u* and *v*. Consequently, 0 *≤ u ≤* 1 and 0 *≤ v ≤* 1 hold. See Figure 6 for an area Type A, and Figure 7 where it is explained for each area type around a point *m* addressed with integer triplet with sum 1.

Now, the most important properties of Ω will be recalled, details can be found in [15].

**Lemma** **1.** *The sum of the triplet of each point, in *Ω*, is in the closed interval *[−1,1]*. Moreover, the points are classified based on the sum of their coordinate triplets as follows. If the sum is*

- equal to *1*, then it is the midpoint of a positive trixel;
- equal to *−1*, then it is a midpoint of a negative trixel;
- equal to *0*, then the point is on the edge of a trixel;
- positive, then the point belongs to a positive trixel;
- negative, then the point belongs to a negative trixel.

**Lemma** **2.** Every point in the triangular plane has at least one integer value in its triplet. Moreover, the place of the integer value indicates its area (*A*, *B*, or *C*) as follows. The 1st coordinate value of every point in Area *A* is the same as the 1st coordinate value of the midpoint. The 2nd coordinate value of every point in Area *B* equals the 2nd coordinate value of the midpoint. Similarly, the 3rd coordinate value of any point in Area *C* equals the 3rd coordinate value of the midpoint. If a triplet contains two integer values, then the point is located on the line of the border between two different type areas. However, if three integers are in a triplet, then this triplet addresses either a midpoint or a vertex (corner) of a trixel.

**Figure 8:**
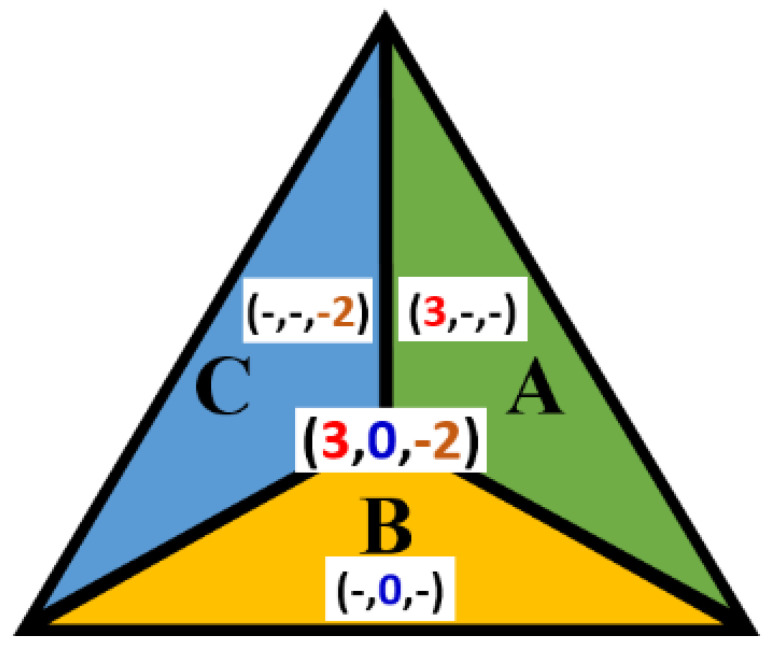
The corresponding constant coordinate value for each area.

Figure 8 shows an example for a trixel and its regions. As another example, consider a triplet of the form (1, 0, *k*). It addresses a point on the line (side of the obtuse-angled triangle) between Areas A and B (0 ≤ *k* ≤ −1).

In the next subsection, we will recall the conversion between Ω and the Cartesian coordinate system.

### 2.3. Converting Triplets to Cartesian Coordinates and Vice Versa

In Ω, axes *I, J*, and *K* are used to describe the triangular plane (Figure 2b), consequently, triplet (*i, j, k*) is used to identify a point in Ω. The usual notation (*x, y*) is used to indicate a point in the Cartesian plane, based on coordinate axes *X* and *Y*. The side-length of the trixel of the triangular grid is fixed to be $\sqrt{3}$, and the height is 1.5. Then, Equation (1) is used to compute the corresponding coordinate values *x* and *y* for the given triplet (*i*, *j*, *k*). The conversion is recalled from [15].

**Lemma** **3.** *For any triplet (i, j, k) of the continuous coordinate system, the Cartesian (x, y) is computed by*

(1) $$\left(\begin{array}{ccc} \frac{\sqrt{3}}{2} & 0 & -\frac{\sqrt{3}}{2}\\ \frac{1}{2} & -1 & \frac{1}{2} \end{array}\right)\cdot \left(\begin{array}{l} i\\ j\\ k \end{array}\right)=\frac{1}{2}\cdot \left(\begin{array}{c} \sqrt{3}(i-k)\\ i-2j+k \end{array}\right)=\left(\begin{array}{l} x\\ y \end{array}\right)$$

Now, the conversion to the inverse direction is shown.

**Figure 9:**
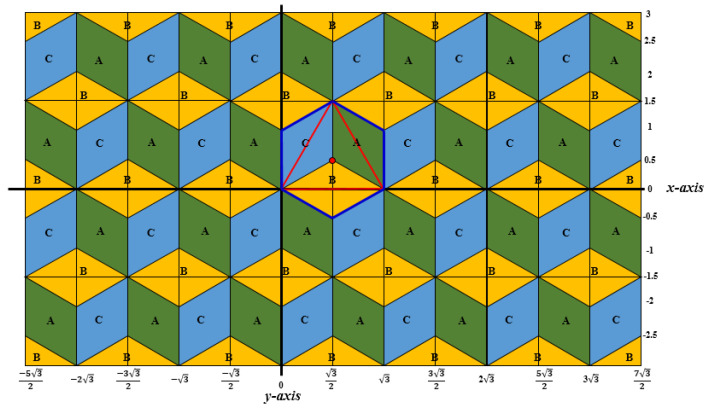
The plane is tessellated by three types of rhombuses; it can also be seen as the surface of a mesh of the cubic grid having three faces of each cube on the surface.

The whole plane built up by rhombuses of Types A, B and C as it can be seen in Figure 9. The conversion of the Cartesian coordinates to equivalent triplets of Ω depends on the type of the area where the point (*x*, *y*) lies.

**Lemma** **4.** Knowing the area where (x, y) belongs, the appropriate formula of Table 1 is used to compute the continuous coordinate triplet (i, j, k) of the same point. In Area *A*, first coordinate i, then coordinate k and finally coordinate j are computed. In Area *B*, the computation starts with j, then i and k follow. In Area *C*, the sequence of computation starts with k, then continues with i and j.

## 3. Procedure for Adding Vectors in Ω

**Figure 10:**
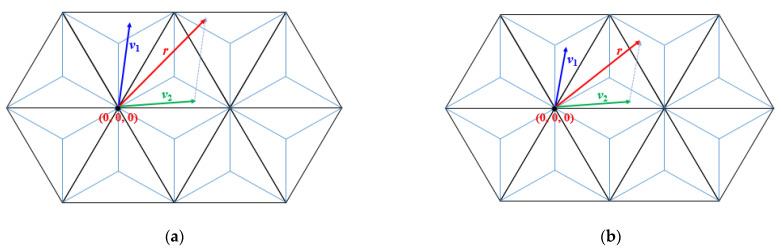
(**a**) Consider vectors *v*_1_ = (0.387, −1, 0.213) and *v*_2_ = (0.677, 0, −0.477); both are Type B. In this case, the direct-sum of vectors will be *s* = (1.064, −1, −0.264), which is Type B as well and hence is a result-vector for Ω. (**b**) Consider vectors *v*_1_ = (0.173, −0.813, 0) and *v*_2_ = (0.677, 0, −0.477) of Types C and B*,* respectively. In this case, the direct-sum of vectors will be *s* = (0.851, −0.813, −0.477), which is not compatible with Ω showing the nonlinearity of the system.

In contrast to the simplicity of vector addition in the Cartesian system (where it is really only an algebraic addition on the coordinate components), the addition in Ω is not so straightforward, i.e., adding two vectors directly (algebraically) may give an improper vector to Ω (see Figure 10a,b). Therefore, some modifications on the direct-sum (*s*) of the vectors are needed to get the result-vector (*r*), which is compatible with Ω. As direct addition of the vectors do not work in general, one can see that our (coordinate) system is not linear. Actually, as Figure 9 shows we may consider it in a way that the points of the Euclidean plain are mapped to a cubic mesh surface built up by faces of the cubes.

Let us consider two vectors *v*_1_ = (*i*_1_, *j*_1_, *k*_1_) and *v*_2_ = (*i*_2_, *j*_2_, *k*_2_) of Ω with their coordinate triplets. Let the algebraic, direct-sum of the vectors be *s* = (*i*, *j*, *k*) = (*i*_1_ + *i*_2_, *j*_1_ + *j*_2_, *k*_1_ + *k*_2_). This vector may not represent any point of Ω. To describe our method and formula mathematically, we shall introduce some more notions and notations.

### 3.1. Further Definitions and Notations

The coordinate values of each vector are real numbers, having some integer and fractional parts. We use the following description for them.

Each coordinate value *x* consists of:

(1) ⟦x⟧: The integer part of *x* with sign, i.e.;
  $⟦x⟧=sgn\left(x\right)\cdot \;\left\lfloor |x|\right\rfloor \;=sgn\left(x\right)\langle |x|-\frac{1}{2}\rangle$, where
  $$sgn\left(x\right)=\;\{\begin{array}{c} \;+\;1,\;x>0\\ 0,\;x=0\\ -1,\;x<0 \end{array}$$
  and ⌊x⌋ is the floor function, that is applied on the absolute value of *x* above. One may also use the rounding operation that we have already defined in Table 1.
(2) {*x*}: The fractional part of *x* with sign, i.e.,
  {x}=x−⟦x⟧
(3) ⌊x⌉: The absolute-rounding-up operation that rounds a number up in absolute value, such that:
  $$\lfloor x\rceil =⟦x⟧+\;sgn\left(\left\{x\right\}\right)=sgn\left(x\right)\langle \left|x\right|\;+\;\frac{1}{2}\rangle .$$

**Example** **1.**

- ⟦1.3⟧ = 1 *and* ⟦−1.3⟧ = −1
- {1.3} = 0.3 *and* {−1.3} = − 0.3
- ⌊1.3⌉ = 2 *and* −⌊0.4⌉ = −1

**Figure 11:**
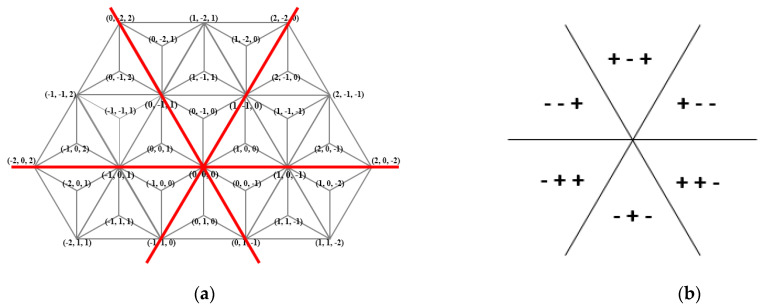
(**a**) The six regions of the triangular plane. (**b**) The signs of the coordinate triplet for each region of the triangular plane.

In order to have a valid addition of two vectors in Ω producing correct result in Ω, some calculations must be done. In these calculations, the following two notions, the *Rsum* and the region, must be defined first:

(1) Rsum= ⌊i⌉ + ⌊j⌉ + ⌊k⌉, where *i*, *j*, and *k* are the coordinate values of the direct-sum *s*.
(2) The region is based on the signs of the coordinates of the direct-sum. Accordingly, the triangular grid is partitioned into six regions based on the signs of the coordinates of the triplet used in the region, as it is shown in Figure 11a,b. The region can be specified by checking the three coordinate values of *s* as follows. For each coordinate value, *x*, of the direct-sum vector, the sign is described as
  (2) $$\mathrm{sign}\;\mathrm{of}\;x=\;\{\begin{array}{c} +,\;\mathrm{if}\;x>0,\;\mathrm{or}\;x=0\;\mathrm{and}\;sum\leq 0,\\ -,\;\mathrm{if}\;x<0,\;\mathrm{or}\;x=0\;\mathrm{and}\;sum>0, \end{array}$$
  where *sum* is the sum of the three coordinate values of direct-sum vector, i.e., *sum* = *i + j* + *k*.

### 3.2. The Algorithm for the Computation

Our calculations are given in the algorithmic form. The procedure below computes the result of the addition of the two input vectors. The computation is based on the types of the two added vectors (the areas A, B, or C they belong to) and some features of the direct-sum *s* = (*i*, *j*, *k*).
**Procedure** to find the result-vector *r* Input: *v*_1_ = (*i*_1_, *j*_1_, *k*_1_) and *v*_2_ = (*i*_2_, *j*_2_, *k*_2_), two vectors of Ω. Output: *r* = (*i*, *j*, *k*) = *v*_1_ + *v*_2_, the result-vector in Ω. Step (1) Let:     (a) direct-sum *s: =* (*i*, *j*, *k*) = (*i*_1_ + *i*_2_, *j*_1_ + *j*_2_, *k*_1_ + *k*_2_)     (b) Rsum:= ⌊i⌉ + ⌊j⌉ + ⌊k⌉    (c) Region: be the signs of the triplet of direct-sum. Step (2) Find the type of the result-vector (*r*):If (*v*_1_ type = *v*_2_ type)Then, the type of *r* is determined based on the *Rsum* value and the comparison of {$\bar{i}$}, {$\bar{j}$}, and {$\bar{k}$} values. (*Explained later, see Equation (3) and Table 3.*)
If (*v*_1_ type ≠ *v*_2_ type)Then, the type of *r* is determined based on the *Rsum* value and the *minimum* (*maximum*) of ({$\bar{i}$}, {$\bar{j}$}, and {$\bar{k}$} values. (*Explained later, see Table 3.*)
 Step (3) Calculate the result-vector (*r*) by applying the following two steps:
(a)Switch (*r* type):case A: let *r*: = (*i* − ({*i*_1_} + {*i*_2_}), *j* − ({*i*_1_} + {*i*_2_}), *k* − ({*i*_1_} + {*i*_2_}))case B: let *r*: = (*i* − ({*j*_1_} + {*j*_2_}), *j* − ({*j*_1_} + {*j*_2_}), *k* − ({*j*_1_} + {*j*_2_}))case C: let *r*: = (*i* − ({*k*_1_} + {*k*_2_}), *j* − ({*k*_1_} + {*k*_2_}), *k* − ({*k*_1_} + {*k*_2_}))
(b)If ((*i + j + k*) *>* 2) Then, let *r*: = (*i* − 1, *j* − 1, *k* − 1)   Else If ((*i + j + k*) *<* −2) Then, let *r*: = (*i* + 1, *j* + 1, *k* + 1).

This procedure contains three steps. The first two steps are used to gain only the type of the result-vector *r* (A, B or C), whereas the last step, the third one, is for calculating the value of the result-vector (*r*). The result-vector (*r*) will be the final correct vector, while the direct-sum (*s*) will be the vector that is generated by direct addition, which is in many cases, incompatible with Ω. In the next subsections, we will explain the above procedure in detail.

### 3.3. Step 1: Finding the Direct-Sum, Rsum, and the Region

The first step of this procedure is to find the direct-sum *s*, the *Rsum* value, and the region, as mentioned above.

For simplicity, only the region of (+, −, −) is considered in the next description, while for all other regions similar calculation methods are applied.

Now, regarding the *Rsum* value, note here that whenever we add two vectors of the same type, at the region (+, −, −), the value of *Rsum* is in the set {−1, −2, 0}. More precisely, within the three coordinate values of each vector, one is an integer value; thus, actually, the fractional parts of the other two coordinate values are responsible for producing one of the three values of the set above.

For a better explanation, consider Table 2, where Samples (*a*), (*b*), and (*c*) have additions of vectors, Type A. In Sample (*a*), the addition of the 2nd coordinate value is the only one that carries 1, therefore, *Rsum* = −1 is produced, while for Sample (*b*), the addition of the 2nd and 3rd coordinate values carries 1, thus *Rsum* = −2, whereas the addition of Sample (*c*) does not lead to carrying 1, hence *Rsum* = 0. Moreover, Samples (*a*), (*b*), and (*c*) generate the three possible values (−1, −2, and 0) of *Rsum* for the addition of two vectors of Type A at this region, (+, −, −). Carry in the direct addition may occur on the coordinates which have a nonzero fractional part in both input vectors *v*_1_ and *v*_2_.

In contrast, the addition of two vectors of different types at this region, (+, −, −), will produce only two possible values of *Rsum* from the set {−1, 0}, where at most one carry could be occurred in these cases. (See Table 2, Samples (d) and (e).)

### 3.4. Step 2: Finding the Type of the Result-Vector

As mentioned above, adding two vectors of the same type would produce any of the three possible values of *Rsum*. Only one of these values would lead directly to the type of the result-vector, while for the other two values one needs more steps to find it. However, the values of *Rsum* that lead directly to the type of the result-vector, in the region (+,−,−), are the following:

- If (adding vectors of types (A + A)) and (*Rsum* = −1)
  then the result-vector type is A.

- If (adding vectors of types (B + B)) and (*Rsum* = 0)
  then the result-vector type is B.

- If (adding vectors of types (C + C)) and (*Rsum* = 0)
  then the result-vector type is C.

In order to demonstrate the three cases above, let us consider the first one, while the other two points would have a similar demonstration.

Assume that *a* = (*i*, *j*, *k*) is the midpoint of a positive triangle. Let *P* be any point (vector) in Area A with corner point *a*. Then, by the barycentric equation we have:

*P* = *a* + *v* · (*b* − *a*) + *u* · (*c* − *a*)

The values of (*b* − *a*) and (*c* − *a*) are fixed for all points in the given Area A (see also Figure 6b), hence we have:

$$P=\left(\begin{array}{l} i\\ j\\ k \end{array}\right)+v\left(\begin{array}{c} 0\\ -1\\ 0 \end{array}\right)+u\left(\begin{array}{c} 0\\ 0\\ -1 \end{array}\right)=\left(\begin{array}{c} i\\ j-v\\ k-u \end{array}\right)$$

Now, Rsum= ⌊i⌉ + ⌊j−v⌉ + ⌊k−u⌉

Since the region of (+, −, −) is considered here, then:

⌊i⌉: Since *i* is a positive integer, then ⌊i⌉ equals to *i* itself.

⌊j−v⌉: Since *j* is a negative integer, then ⌊j−v⌉ equals to *j* − 1.

⌊k−u⌉: Since *k* is a negative integer, then ⌊k−u⌉ equals to *k* − 1.

Then *Rsum* = *i* + (*j* − 1) + (*k* − 1), but since (*i*, *j*, *k*) is a positive midpoint then *i* + *j* + *k* = 1, hence, *Rsum* = −1. Therefore, two vectors of Type A would produce a new vector of Type A if and only if the *Rsum* value is equal to −1.

The type of the result-vector could be one of the three possible types (A, B or C). We have already seen one of the possibilities, it has been obtained for a particular *Rsum* value, and the remaining two possibilities are going to be determined in the next part.

Before determining the other possibilities, it is worth mentioning here that, since every direct-sum has one value among its three coordinates with a different sign, based on the region signs, it is inconvenient to use logical comparison operations (<, >, min or max) among the values {*i*}, {*j*}, and {*k*}. (There is also a kind of imprecision by a lack of agreement in mathematics, programming and various software or calculators to compute fractional parts of negative numbers, e.g., the fractional part of −0.35 can be 0.35, or −0.35, or 0.65 by using various approaches (various software). Thus, to make it clear how our computation process is going, we give some technical details to avoid the mentioned ambiguity.) One of the “technical tricks” used in our procedure is to unify these signs, just for the comparison purpose, such that all of them must be converted to one of the two signs, either all positive or all negative. Here, since we are describing the region (+, −, −), we will convert those fractional parts that have zero or positive factional value into negative values by subtracting 1 from them. Formally, we can introduce the following technical notation:

(3) $$\left\{\bar{i}\right\}=\{\begin{array}{c} \left\{i\right\}\\ \left\{i\right\}-1 \end{array}\;\begin{array}{c} \mathrm{if}\;\left\{i\right\}<0,\\ \mathrm{otherwise}. \end{array}\;$$

In a similar manner, notations {$\bar{j}$} and {$\bar{k}$} for the fractional parts {*j*} and {*k*} are also used to guarantee that the conversion is utilized to unify the signs if needed. See also Example 2. It is also possible to convert the negative fractions to positive ones, but in this explanation we use negative fractions. Clearly, in this way, integers have the smallest fractional part, and we can really compare the values without having problem caused by the signs in various software tools as we have already mentioned; the result does not depend on the used programming and software environment. For the sake of clarity we also provide a simple example.

**Example** **2.** *If a positive fractional part* {i}=0.35 *, then it will be converted to* $\left\{\bar{i}\right\}=-0.65$ *as* 0.35 − 1 = −0.65. *In addition, zero as a fractional part will be converted to* −1, *since* 0 − 1 = −1.

Therefore, whenever a comparison operation is applied, we use the fraction values with united negative sign {$\bar{i}$}, {$\bar{j}$}, and {$\bar{k}$}.

Now, *Rsum* values are in the set {−2, −1, 0} and *Rsum* = −1 leads directly to have a result-vector of Type A. If *Rsum* = 0 and {$\bar{j}$} < {$\bar{k}$}, then the result-vector is of Type B, otherwise it is of Type C. If *Rsum* = −2 and {$\bar{j}$} > {$\bar{k}$}, then the result-vector is of Type B, otherwise it is of Type C. Note here that only {*j*} and {*k*} are considered but not {*i*} because the two added vectors are of Type A.

If vectors of different types were added, apart from the *Rsum* value, the maximum (or minimum) value among some values related to {*i*}, {*j*}, and {*k*} will also be evaluated and thus the type of the result-vector would be specified. In order to specify the result-vector type in all other cases, see Table 3.

Note here that when applying comparison operation on equal values then selecting any of the given types would be correct, because the point is on a border line or it is a vertex.

### 3.5. Step 3: Finding Coordinate Triplet of the Result-Vector

Once the type of the result-vector has been determined, we proceed to Step 3. Where part (*a*) has three possibilities based on the type of the result-vector, as follows.

- If the type of the result-vector is A, then its coordinate triplet is:
  *r* = (*i* − ({*i*_1_} + {*i*_2_}), *j* − ({*i*_1_} + {*i*_2_}), *k* − ({*i*_1_} + {*i*_2_})),

where {*i*_1_} and {*i*_2_} are the fractional parts of the first coordinate value of the first and second vectors, respectively.

- If the type of the result-vector is B, then its coordinate triplet is:
  *r* = (*i* − ({*j*_1_} + {*j*_2_}), *j* − ({*j*_1_} + {*j*_2_}), *k* − ({*j*_1_} + {*j*_2_})),

where {*j*_1_} and {*j*_2_} are the fractional parts of the second coordinate value of the first and second vectors, respectively.

- If the type of the result-vector is C, then its coordinate triplet is:
  *r* = (*i* − ({*k*_1_} + {*k*_2_}), *j* − ({*k*_1_} + {*k*_2_}), *k* − ({*k*_1_} + {*k*_2_})),

where {*k*_1_} and {*k*_2_} are the fractional parts of the third coordinate value of the first and second vectors, respectively.

Eventually, part (*b*) of this step, is about subtracting or adding 1 from/to each coordinate value, is applied if their sum is greater than 2 or less than −2, respectively.

**Theorem** **1.** *The Procedure is correct, for any two vectors z = (i_1_, j_1_, k_1_), w = (i_2_, j_2_, k_2_)* ∈ Ω *it produces the vector r = (i_3_, j_3_, k_3_) = z + w* ∈ Ω.

**Proof.**  The proof has been moved to the Appendix A for better readability. □

To display also the applicability of our result, we show also a detailed example.

**Example** **3.** *In* Table 4, *which includes three samples to show different cases, we show examples. To give a detailed view, consider Sample* (*a*) *of* Table 4 *where we have:*

*v*_1_ = (1.577, −2.0, 0.423)

*v*_2_ = (1.005, 0.0, −1.305)

By converting *v*_1_ and *v*_2_ into the Cartesian coordinates *c*_1_ and *c*_2_, using Equation (1), we have:

For *c*_1_ = $\left(\begin{array}{ccc} \frac{\sqrt{3}}{2} & 0 & -\frac{\sqrt{3}}{2}\\ \frac{1}{2} & -1 & \frac{1}{2} \end{array}\right)\cdot \left(\begin{array}{c} 1.577\\ -2.0\\ 0.423 \end{array}\right)=\left(\begin{array}{l} 0.999\\ 3.000 \end{array}\right)$

Then *c*_1_ = (*x*_1_, *y*_1_) = (0.999, 3.000) is the value of *v*_1_ with Cartesian coordinates, and

for *c*_2_ = $\left(\begin{array}{ccc} \frac{\sqrt{3}}{2} & 0 & -\frac{\sqrt{3}}{2}\\ \frac{1}{2} & -1 & \frac{1}{2} \end{array}\right)\cdot \left(\begin{array}{c} 1.005\\ 0.0\\ -1.305 \end{array}\right)=\left(\begin{array}{c} 2.001\\ -0.150 \end{array}\right)$

Then *c*_2_ = (*x*_2_, *y*_2_) = (2.001, −0.150) is the Cartesian vector that corresponds to *v*_2_.

Now, applying the Cartesian addition to *c*_1_ and *c*_2_, we have:

*c =* (*x*, *y*) = *c*_1_ + *c*_2_ = (3.000, 2.850) in Cartesian coordinates

Finally, convert *c* = (*x*, *y*) into triplet in Ω,

*c* belongs to the 1st quarter, and

it belongs to Area B.

Thus, formulae of Area B from Table 1 are applied in the following order:

(1) *j* = $\;\langle \frac{-2y}{3}\rangle$ = −2.0
(2) *i* = $\frac{x\sqrt{3}}{3}\;+\;y\;+\;j$ = $\sqrt{3}$ + 2.85 − 2.0 = 2.582
(3) *k* = $i-\frac{2x}{\sqrt{3}}$ = 2.582 − 3.464 = −0.882

Then the corresponding triplet of (3.000, 2.850) is *r* = (2.582, −2.000, −0.882), which is exactly the same answer our procedure gives in Table 4 as it should be.

We close this section by the following remarks.

Notice that our procedure to add two vectors has a constant time complexity, thus it can be used very efficiently in any type of computation, where some grid points of the triangular grid may be transformed outside of the grid.

## 4. Vector Arithmetic and its Application

In this section our aim is twofold: first, we apply the previous results to give a more general vector arithmetic system; and second, we present a real-life application scenario of our newly investigated system.

### 4.1. Subtraction and Scalar Product

Whenever, one is able to add two vectors, it is straightforward to apply again addition on the result vector with some other vectors, thus by applying the addition scheme for two vectors, as we have described, one can add any finite number of vectors.

Regarding the subtraction operation, we have the following:

**Lemma** **5.** In *Ω*, for any vector v = (i,j,k) its opposite is −v = (−i, −j, −k), such that v + (−v) = *(0,0,0)*.

**Proof.**  Based on Table 1, using −*x* and −*y* instead of *x* and *y*, one may observe that every formula for the coordinate triplet gives −1 times its original value, consequently, if (*x*,*y*) is transformed to (*i*,*j*,*k*), then (−*x*, −*y*) is transformed to (−*i*, −*j*, −*k*) completing the proof. □

**Theorem** **2.** *Let v_1_, v_2_*∈ Ω*, the subtraction v_1_ − v_2_ is computed as the vector addition v_1_ + (−v_2_).*

**Proof.**  To see that the formula is correct, one needs to apply Lemma 5. □

Finally, we investigate scalar product with integer multiplier.

**Theorem** **3.** *Let v* **∈ Ω* and n be an integer. The following procedure will provide the vector r = n* *⋅ v.*

Input: *v* = (*i*, *j*, *k*) and integer *n.*
Output: *r* = (*i_r_*, *j_r_*, *k_r_*) the scalar product of the number *n* and vector *v*.
Step1) If *n* = 0 then let *r*: = (0,0,0). Stop.
Step2) If *n* < 0 then let *n*: = − *n* and *v*: = − *v*.
Step3) Let *r*: = *v*.
Step4) If *n* = 1 then Stop.
Step5) For *h* = 2 to *n* do
Step6) Let *r*: = *r* + *v*.
Step7) Endfor.
Step8) Stop.

**Proof.**  Step 1 gives the answer as the null-vector in obvious case. If the coefficient *n* is negative, the solution is computed based on the identity −*m* ⋅ *v* = *m* ⋅ (*−v*), where *m* is a non-negative integer, based on Lemma 5 and Theorem 2. Whenever, *n* = 1 or *n* = −1 we do not need to do any addition, the result is either same as the original value *v* or it is −*v*. Vector addition is iterated in the for loop if |*n*| ≥ 2 till the final result is obtained in the vector variable *r*. □

**Figure 12:**
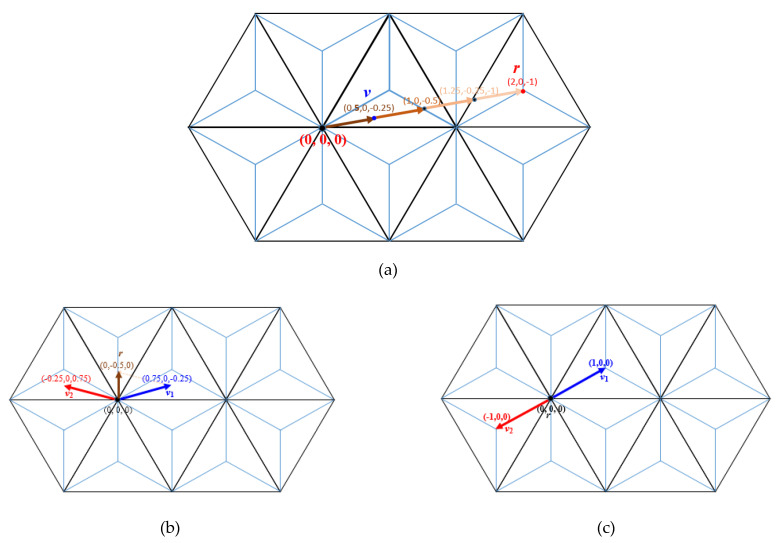
Examples for vector arithmetic: (**a**) Consider the scalar product of vector *v* = (0.5, 0, −0.25) with a positive integer multiplier *n* = 4 (that is to compute *v + v + v + v*) which yields to vector *r* = (2, 0, −1). (**b**) Consider the addition of vectors *v*_1_ = (0.75, 0, −0.25) and *v*_2_ = (−0.25, 0, 0.75) which results in vector *r* = (0, −0.5, 0). (**c**) The addition of the opposite vectors *v*_1_ = (1, 0, 0) and *v*_2_ = (−1, 0, 0) give vector *r* = (0, 0, 0).

**Example** **4.** Figure 12 *shows some applications: In* Figure 12a, *e.g., the scalar products* 3 ⋅ (0.5, 0, −0.25) = (1.25, −0.25, −1) *and* 4 ⋅ (0.5, 0, −0.25) = (2, 0, −1) *are shown. The vector subtraction* (0, −0.5, 0) − (−0.25, 0, 0.75) = (0.75, 0, −0.25) *which can also be interpreted as vector addition* (−0.25, 0, 0.75) + (0.75, 0, −0.25) = (0, −0.5, 0) *is shown in* Figure 12b. *Finally,* Figure 12c *shows* −1 ⋅ (1, 0, 0) = (−1, 0, 0) *and also as* (1, 0, 0) + (−1, 0, 0) = (0, 0, 0).

### 4.2. Application: Drawing and Imaging on a Cubic Mesh

Let us consider the three-dimensional space built up by unit cubes. Considering a mesh with norm (1,1,1) we got an oblique plane which is closely related to the hexagonal and triangular grids considering only the grid-points [38]. However, considering the whole surface of the square faces of the cubes which are on the boundary, a three-dimensional surface is obtained which has a one-to-one correspondence with the continuous triangular plane with the coordinate triplets, somewhat similarly as the image of Figure 9 can be seen as such a cubic mesh. The vector arithmetic we have studied here fits well to this surface, because the coordinate systems are identical. Thus, to draw on this surface, one can compute which points should have what color based on our results. In addition, if an image is drawn in this surface, to capture it and/or to do any modifications, e.g., translation, or enlarging (zoom in) it to double size, our results can efficiently be used.

**Figure 13:**
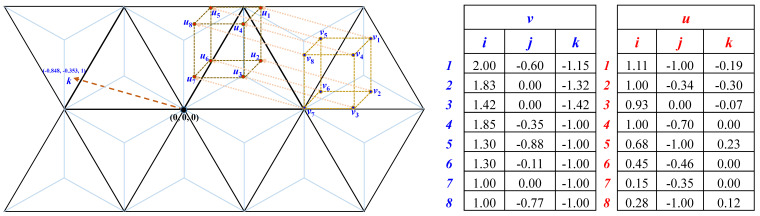
Example of image translation by vector addition: the points represented by vectors *v*_1_, …, *v*_8_ have been translated by vector *k* = (−0.848, −0.353, 1) to get the points represented by vectors *u*_1_, …, *u*_8_.

**Example** **5.** *Let an image be on the cubic mesh surface that contains the points v*_1_,…,*v*_8_ (*shown by blue color in* Figure 13). *Then, translating this image by the vector k* = (−0.848, −0.353, 1), *the points u*_1_, …, *u*_8_ (*shown by red color in* Figure 13) *are obtained to form the translated image.*

**Figure 14:**
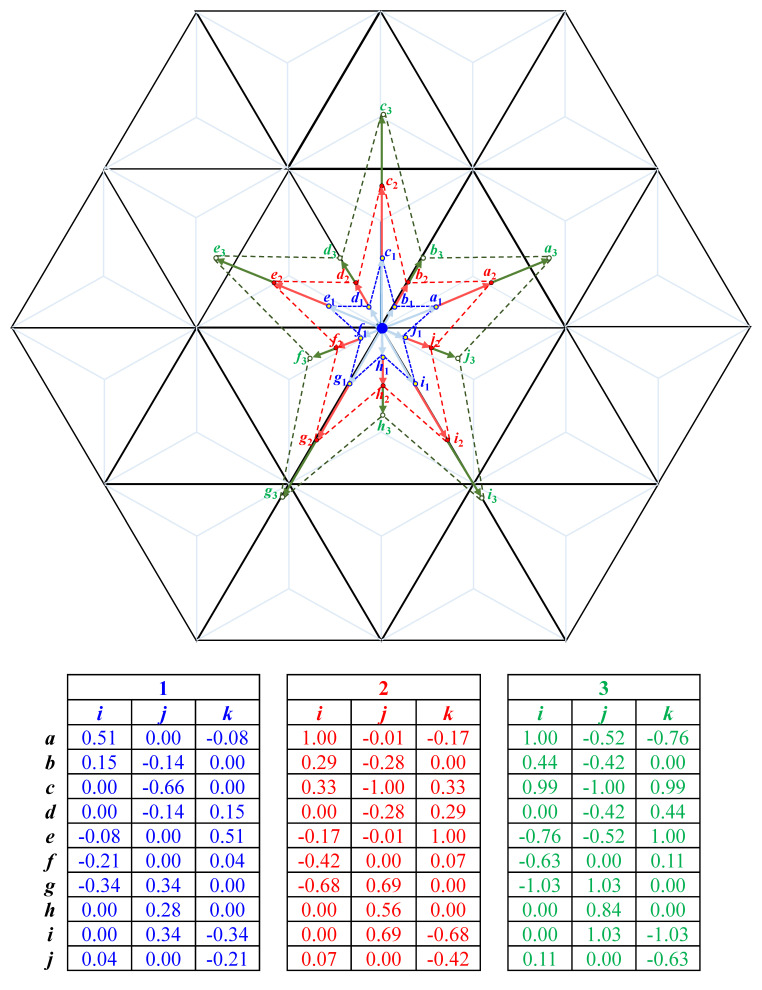
Example for zooming an image by vector arithmetic: the star defined by points represented by vectors *a*_1_, …, *j*_1_ given in blue color are doubled and tripled, the resulted vectors and shapes are presented in red and green color.

**Example** **6.** *Let a star shape image be given by the points a*_1_, …, *j*_1_, *as it is shown in* Figure 14 *by blue color. The double and triple sized images can be computed by the scalar product of the vectors addressing the points a*_1_, …, *j*_1_ *by scalar* 2 *and* 3, *respectively. The obtained points are shown by red and green color with Indices* 2 *and* 3 *with their coordinate values in the figure*.

## 5. Conclusions

The discrete symmetric coordinate systems for the triangular grid have been extended to the continuous coordinate system. In this paper vector addition, subtraction and scalar product (with integer coefficients) are studied in this system showing that although vector arithmetic is more complex in this grid than on usual point-lattices, e.g., on rectangular grids, one can manage it with short and efficient codes (with some formulae depending on various cases described by a set of conditions). As a first possible real-life application, we shown how drawing and imaging can be done on the non-linear surface of a mesh on the cubic grid where the points of the square surfaces of the cubes on the boundary can be addressed by our continuous coordinate system. However, this symmetric coordinate system is indeed very helpful for various applications concerning the triangular grid, where the grid points are not necessarily mapped to grid points, e.g., arbitrary angled rotations, zooming or interpolation of images. This coordinate system provides a new tool: on the one hand it can be converted to/from the Cartesian coordinate system, but, more importantly, on the other hand, with the vector arithmetic presented here, it can directly be used to transform images of the triangular plane. Vector arithmetic including vector addition is very fundamental tool for working with images. By extending our work with a digitization/re-digitization which is mapping every point of a triangle pixel to the midpoint of the triangle by identifying the trixel where the point belongs, our system is ready to use for research and applications of digital images on the triangular grid such as imaging, computer graphics, image manipulation, image processing and analysis including various applications e.g., transformations and other operations that do not necessarily map the grid to itself. The presented and used coordinate system is continuous and, therefore, there is no information loss with isometric transformations, similarly to the case when the entire plane is addressed by the Cartesian coordinate frame. The information loss which is an important factor at various transformations, e.g., rotations of digital images is coming when the result is re-digitized, which operation, of course, causes certain information loss in many cases either or both on the square and on the triangular grids. Some preliminary studies on the digital rotations comparing these grids can be found in [40].

It is a topic of future work to find a short way to compute also scalar product with fractional values.

## Figures and Tables

**Table 1:** Formulae for the coordinate triplets based on the type of the area. The operation 〈…〉 is a rounding operation *.

	Area A	Area B	Area C
***i***	$\langle \frac{x}{\sqrt{3}}\rangle +\langle \frac{y}{3}\rangle$	$\frac{x\sqrt{3}}{3}+y+j$	$\frac{2x}{\sqrt{3}}+k$
***j***	$\frac{i+k}{2}-y$	$\langle \frac{-2y}{3}\rangle$	$\frac{i+k}{2}-y$
***k***	$i-\frac{2x}{\sqrt{3}}$	$i-\frac{2x}{\sqrt{3}}$	$\langle \frac{y}{3}\rangle -\langle \frac{x}{\sqrt{3}}\rangle$

* Rounding operation returns the nearest integer to the real number, such that numbers exactly the same distance from two integers are rounded to the larger absolute valued one, e.g., 〈2.5〉=3, 〈−2.5〉=−3 and 〈−0.35〉=0.

**Table 2:** Different samples to demonstrate the first step of the procedure (boldface coordinate values produce carry values).

	Sample (a)	Sample (b)	Sample (c)	Sample (d)	Sample (e)
**Vector1**	(1.0,**−0.6**,−0.1)	(1.0,**−0.6,−0.3**)	(1.0,−0.6, −0.1)	(1.0,−0.9,**−0.4**)	(1.0,−0.9,−0.4)
**Vector2**	(1.0,**−0.9**,−0.7)	(1.0,**−0.9,−0.7**)	(1.0,−0.2,−0.7)	(1.9,−1.0,**−0.9**)	(1.9,−1.0,−0.5)
**direct-sum**	(2.0,−1.5,−0.8)	(2.0,−1.4,−1.09)	(2.0,−0.8,−0.8)	(2.9,−1.9,−1.3)	(2.9,−1.9,−0.9)
⌊i⌉, ⌊j⌉,⌊k⌉	2, −2, −1	2, −2, −2	2, −1, −1	3, −2, −2	3, −2, −1
**Rsum**	**−1**	**−2**	**0**	**−1**	**0**
**Region**	(+, −, −)	(+, −, −)	(+, −, −)	(+, −, −)	(+, −, −)

**Table 3:** All conditions and rules for specifying the type of result-vector.

**Regions**	**Vectors of Type (A + A)**
(−, −, + ) (−, +, −) (+, −, + ) (+, +, −)	IF (*Rsum* = 0) THEN result-vector type is A
	IF (*Rsum* = 1) & ({$\bar{j}$}≤{$\bar{k}$}) THEN result-vector type is B ELSE C
	IF (*Rsum* = *−*1) & ({$\bar{j}$}≥{$\bar{k}$}) THEN result-vector type is B ELSE C
(−, +, + )	IF (*Rsum* = 1) THEN *result-vector* type is A
	IF (*Rsum* = 0) & ({$\bar{j}$}≥{$\bar{k}$}) THEN result-vector type is B ELSE C
	IF (*Rsum* = 2) & ({$\bar{j}$}≤{$\bar{k}$}) THEN result-vector type is B ELSE C
(+, −, −)	IF (*Rsum* = *−*1) THEN result-vector type is A
	IF (*Rsum* = 0) & ({$\bar{j}$}≤{$\bar{k}$}) THEN result-vector type is B ELSE C
	IF (*Rsum* = *−*2) & ({$\bar{j}$}≥{$\bar{k}$}) THEN result-vector type is B ELSE C
**Regions**	**Vectors of type (B + B)**
(−, +, + ) (−, −, + ) (+, −, −) (+, +, −)	IF (*Rsum* = 0) THEN result-vector type is B
	IF (*Rsum* = 1) & ({$\bar{i}$}≤{$\bar{k}$}) THEN result-vector type is A ELSE C
	IF (*Rsum* = −1) & ({$\bar{i}$}≥{$\bar{k}$}) THEN result-vector type is A ELSE C
(+, −, + )	IF (*Rsum* = 1) THEN result-vector type is B
	IF (*Rsum* = 0) & ({$\bar{i}$}≥{$\bar{k}$}) THEN result-vector type is A ELSE C
	IF (*Rsum* = 2) & ({$\bar{i}$}≤{$\bar{k}$}) THEN result-vector type is A ELSE C
(−, +, −)	IF (*Rsum* = *−*1) THEN result-vector type is B
	IF (*Rsum* = 0) & ({$\bar{i}$}≤{$\bar{k}$}) THEN result-vector type is A ELSE C
	IF (*Rsum* = 2) & ({$\bar{i}$}≥{$\bar{k}$}) THEN result-vector type is A ELSE C
**Regions**	**Vectors of type (C + C)**
(−, +, + ) (+, −, + ) (+, −, −) (−, +, −)	IF (*Rsum* = 0) THEN result-vector is of type C
	IF (*Rsum* = 1) & ({$\bar{i}$}≤{$\bar{j}$}) THEN result-vector is of type A ELSE B
	IF (*Rsum* = −1) & ({$\bar{i}$}≥{$\bar{j}$}) THEN result-vector is of type A ELSE B
(−, −, + )	IF (*Rsum* = *−*1) THEN result-vector is of type C
	IF (*Rsum* = 0) & ({$\bar{i}$}≤{$\bar{j}$}) THEN result-vector is of type A ELSE B
	IF (*Rsum* = −2) & ({$\bar{i}$}≥{$\bar{j}$}) THEN result-vector is of type A ELSE B
(+, +, −)	IF (*Rsum* = 1) THEN result-vector is of type C
	IF (*Rsum* = 0) & ({$\bar{i}$}≥{$\bar{j}$}) THEN result-vector is of type A ELSE B
	IF (*Rsum* = 2) & ({$\bar{i}$}≤{$\bar{j}$}) THEN *result-vector* is of type A ELSE B
**Regions**	**Vectors of type (A + B) or (A + C) or (B + C)**
(−, −, + ) (−, +, −) (+, −, −)	IF (*Rsum* = 0) THEN Min({$\bar{i}$},{$\bar{j}$},{$\bar{k}$}) is the result-vector type * IF (*Rsum* = *−*1) THEN Max({$\bar{i}$},{$\bar{j}$},{$\bar{k}$}) is the result-vector type *
(+, −, + ) (+, +, −) (−, +, + )	IF (*Rsum* = 0) THEN Max({$\bar{i}$},{$\bar{j}$},{$\bar{k}$}) is the result-vector type * IF (*Rsum* = 1) THEN Min({$\bar{i}$},{$\bar{j}$},{$\bar{k}$})is the result-vector type *

* if {$\bar{i}$}, {$\bar{j}$} or {$\bar{k}$} is the minimum (maximum, resp.) then the result-vector type is A, B or C respectively. If any two or three values of {$\bar{i}$}, {$\bar{j}$} and {$\bar{k}$}, are equal then the result-vector would be on a border or on a vertex which means selecting any type of them would be correct.

**Table 4:** The full procedure to compute the result-vector with different samples. (boldface coordinate values produce carry values).

	Sample (a)	Sample (b)	Sample (c)
***v*_1_ = (*i*_1_*, j*_1_*, k*_1_)**	(1.577, **−2.0**, 0.423)	(1.155, −1.423, **0.0**)	(**1.0**, −0.735, −0.270)
***v*_2_ = (*i*_2_*, j*_2_*, k*_2_)**	(1.005, **0.0**, −1.305)	(0.808, **0.0**, −0.808)	(**1.0**, −0.966, −0.732)
**Step 1**			
**direct-sum**	(2.582, −2.0, −0.882)	(1.963, −1.423, −0.808)	(2.0, −1.701, −1.002)
**Rsum**	3 + (−2) + (−1) = **0**	2 + (−2) + (−1) = **−1**	2 + (−2) + (−2) = **−2**
**Region**	(+, −, −)	(+, −, −)	(+, −, −)
**Step 2**			
**Rule 1 and 2**	If (*Rsum* = 0) Then B	If (*Rsum* = −1) Then Max({$\bar{i}$},{$\bar{j}$},{$\bar{k}$})	If (*Rsum* = −2) & ({$\bar{j}$} ≥ {$\bar{k}$}) Then B else C
**Apply** **Rule 1 and 2**	−	Max ((0.963−1), −0.423, −0.808) = −0.037 = {$\bar{i}$}	(−0.701) ≯ (−0.002)
**Type of Result-vector**	**B**	**A**	**C**
**Step 3**			
**a) *s* =**	(*i* − ({*j*_1_} + {*j*_2_}*,* *j* − ({*j*_1_} + {*j*_2_})*,* *k* − ({*j*_1_} + {*j*_2_}) )	(*i* − ({*i*_1_} + {*i*_2_})*,* *j* − ({*i*_1_} + {*i*_2_})*,* *k* − ({*i*_1_} + {*i*_2_}) )	(*i* − ({*k*_1_} + {*k*_2_})*,* *j* − ({*k*_1_ } + {*k*_2_})*,* *k* − ({*k*_1_} + {*k*_2_}) )
**Apply a) *s* =**	(2.582,**−2.0**,−0.882)	(**1.0**, −2.386, −1.771)	(3.002, −0.699, **0.0**)
**b) Sum =**	Sum = −0.3, then no need for addition or subtraction of 1	Sum = −3.157 < −2, then add 1 to each coordinate value	Sum = 2.303 > 2, then Subtract 1 from each coordinate value
**Result-vector**	(2.582, **−2.0**, −0.882)	(**2.0**, −1.386, −0.771)	(2.002, −1.699, **−1.0**)

## Appendix A

**Theorem** **A1.** *The Procedure is correct, for any two vectors z = (i_1_, j_1_, k_1_), w = (i_2_, j_2_, k_2_)* ∈ Ω *it produces the vector r = (i_3_, j_3_, k_3_) = z + w*∈ Ω.

**Proof.** The correctness of the procedure can be proven formally by computing the result vector in the previously known way, by converting the input vectors to the Cartesian representation (applying Lemma 3), then applying the algebraic, Cartesian addition, and comparing this result to the result obtained by our procedure (based on the cases detailed in Table 3). Here, we do the comparison in the Cartesian form and we also check if vector we obtained by the procedure is a valid vector in Ω. The full proof is very lengthy and goes by the several cases according to Table 3, however, in this formal proof, we show only one particular case to show its flavor, the sum of two Type A vectors is considered when it lies in the region (+, −, −), as other cases are treated similarly.

Let us consider two Type A vectors, *z* = (*i*_1_, *j*_1_, *k*_1_) and *w* = (*i*_2_, *j*_2_, *k*_2_) such that the direct sum *s* = (*i*, *j*, *k*) lies in the region (+, −, −), i.e., we consider the case when *i* = *i_1_* + *i*_2_ > 0, *j* = *j_1_* + *j*_2_ ≤ 0 and *k* = *k*_1_ + *k*_2_ ≤ 0, moreover *i*_1_ and *i*_2_ are integers. On the first hand, by applying Equation (1) of Lemma 3, the Cartesian coordinates *z*’ and *w*’ of the given vectors *z* and *w* are computed as *z*’= $\left(\frac{\sqrt{3}\left(i_{1}-k_{1}\right)}{2},\frac{i_{1}-2j_{1}\;+\;k_{1}}{2}\right)$ and *w*’= $\left(\frac{\sqrt{3}\left(i_{2}-k_{2}\right)}{2},\frac{i_{2}-2j_{2}\;+\;k_{2}}{2}\right)$. Their sum *r*’ = $\left(\frac{\sqrt{3}\left(i_{1}\;+\;i_{2}-k_{1}-k_{2}\right)}{2},\frac{i_{1}\;+\;i_{2}-2j_{1}-2j_{2}\;+\;k_{1}\;+\;k_{2}}{2}\right)$ gives the Cartesian coordinates of the vector we want to compute also in Ω. In fact, it can also be expressed by the coordinates of *s*: *r*’= $\left(\frac{\sqrt{3}\left(i-k\right)}{2},\frac{i-2j\;+\;k}{2}\right)$.

Now, on the other hand, we also compute the result using our procedure and transform it to Cartesian coordinates. Since *z* = (*i*_1_, *j*_1_, *k*_1_) and *w* = (*i*_2_, *j*_2_, *k*_2_) are in Region A, their coordinates can be written (as it is shown e.g., in Figure 7) as *z* = (*i*_1_, *j_m_*_1_−*u*_1_, *k_m_*_1_−*v*_1_) and *w* = (*i*_2_, *j_m_*_2_−*u*_2_, *k_m_*_2_−*v*_2_), where the triplets (*i*_1_, *j_m_*_1_, *k_m_*_1_) and (*i*_2_, *j_m_*_2_, *k_m_*_2_) address the left bottom corner point of the regions of Type A, where the vectors *z* and *w* are lying, respectively (let us assume that both vectors are inside the Type A area, i.e., 0 < *u*_1_, *v*_1_, *u*_2_, *v*_2_ < 1; the proof for the special cases when one or both vectors are on the border of the region goes in analogous way). By the addressing scheme of the coordinate system, we know that *i*_1_ + *j_m_*_1_ + *k_m_*_1_ = 1 and *i*_2_ + *j_m_*_2_ + *k_m_*_2_ = 1. Now, we can consider our procedure. Since *i* is integer, *Rsum* = i + ⌊j⌉  + ⌊k⌉ = $i\;+\;\lfloor j_{m1}-u_{1}\;+\;j_{m2}-u_{2}\rceil \;+\;\lfloor k_{m1}-v_{1}\;+\;k_{m2}-v_{2}\rceil$ and by taking out the integers from the special rounding operator, we get *Rsum* = $i\;+\;j_{m1}\;+\;j_{m2}\;+\;k_{m1}\;+\;k_{m2}-\lfloor u_{1}\;+\;u_{2}\rceil -\lfloor v_{1}\;+\;v_{2}\rceil$ = $2-\lfloor u_{1}\;+\;u_{2}\rceil -\lfloor v_{1}\;+\;v_{2}\rceil$. The value of any or both of $\left\lfloor u_{1}\;+\;u_{2}\rceil ,\lfloor v_{1}\;+\;v_{2}\right\rceil$ is 1 or 2, thus *Rsum* is in {−2,−1,0} in our cases (as it was already explained earlier). Note that in the given region {$\bar{j}$} = {*j*} and {$\bar{k}$} = {*k*} in case *j* and *k* are not integers and the corresponding value is −1 in case a given value is integer, since both these coordinates are negative. We show the proof for the case when also the resulted vector *r* is inside a given region (when it is on the border the proof is analogous), and in these cases {$\bar{j}$} = {*j*} and {$\bar{k}$} = {*k*}. There are three subcases according to the value of Rsum and the values of {$\bar{j}$} and {$\bar{k}$}.

- By our procedure, if *Rsum* = −1, then vector *r* is of Type A and since *i* is integer, in fact, it is *r* = *s* = (*i*, *j*, *k*). If *Rsum* = −1, then either ($\lfloor u_{1}\;+\;u_{2}\rceil =1$ and $\lfloor v_{1}\;+\;v_{2}\rceil =2$), or ($\lfloor u_{1}\;+\;u_{2}\rceil =2$ and $\lfloor v_{1}\;+\;v_{2}\rceil =1$). Equivalently, ($u_{1}\;+\;u_{2}\leq 1$ and $v_{1}\;+\;v_{2}>1$) or ($u_{1}\;+\;u_{2}>1$ and $v_{1}\;+\;v_{2}\leq 1$). In both cases, *i* + *j* + *k* = $i\;+\;j_{m1}\;+\;j_{m2}\;+\;k_{m1}\;+\;k_{m2}-\left(u_{1}\;+\;u_{2}\right)-\left(v_{1}\;+\;v_{2}\right)=2-\left(u_{1}\;+\;u_{2}\right)-\left(v_{1}\;+\;v_{2}\right)$, and thus 1 ≥ *i* + *j* + *k* ≥ −1 holds, thus no correction is needed. As this triplet is converted exactly to *r*’= $\left(\frac{\sqrt{3}\left(i-k\right)}{2},\frac{i-2j\;+\;k}{2}\right)$, this result matches, proving that our procedure gives the sum correctly in the case when the sum is also a Type A vector.
  To complete the proof of this subcase, we check also that the vector (*i*, *j*, *k*) is valid in Ω. Thus, let us prove this part by converting the Cartesian coordinates of *r*’ to the corresponding triplet coordinates. Let us compute the coordinate triplet *r* = (*l*, *m*, *n*) assigned to *r*’. From the first column of Table 1, the values of *l*, *n* and *m*, in this order can be determined. The first coordinate *l* = $\langle \frac{\sqrt{3}\left(i-k\right)}{2\sqrt{3}}\rangle \;+\;\langle \frac{i-2j\;+\;k}{6}\rangle$ = $\langle \frac{i-k}{2}\rangle \;+\;\langle \frac{i-2j\;+\;k}{6}\rangle$ = $\langle \frac{i-k}{2}\rangle \;+\;\langle \frac{i\;+\;j\;+\;k}{6}-\frac{j}{2}\rangle$. In the given region *i* is always a positive integer, while *j* and *k* have non-positive values, moreover we can substitute them based on $j=j_{m1}-u_{1}\;+\;j_{m2}-u_{2}$ and $k=k_{m1}-v_{1}\;+\;k_{m2}-v_{2}$ by using the abbreviations $j_{m1}=j_{m1}\;+\;j_{m2}$, $k_{m}=k_{m1}\;+\;k_{m2}$, $u=u_{1}\;+\;u_{2}$ and $v=v_{1}\;+\;v_{2}$. Thus, we have *l* = $\langle \frac{i-k_{m}\;+\;v}{2}\rangle \;+\;\langle \frac{i\;+\;j_{m}\;+\;k_{m}}{6}-\frac{j_{m}}{2}\;+\;\frac{2u-v}{6}\rangle$ = $\left\lfloor \frac{i-k_{m}\;+\;v\;+\;1}{2}\rceil \;+\;\lfloor \frac{i\;+\;j_{m}\;+\;k_{m}}{6}-\frac{j_{m}}{2}\;+\;\frac{2u-v}{6}\;+\;\frac{1}{2}\right\rceil$ by taking into account the signs (both parts are nonnegative) and the meaning of the rounding bracket. We already know that $i\;+\;j_{m}\;+\;k_{m}=2$, therefore *l* = $\left\lfloor \frac{i-k_{m}\;+\;v\;+\;1}{2}\rceil \;+\;\lfloor \frac{2}{6}+\frac{1}{2}-\frac{j_{m}}{2}\;+\;\frac{2u-v}{6}\right\rceil$ = $\left\lfloor \frac{i-k_{m}\;+\;v\;+\;1}{2}\rceil \;+\;\lfloor \frac{5}{6}-\frac{j_{m}}{2}\;+\;\frac{2u-v}{6}\right\rceil$. Since the sum $i\;+\;j_{m}\;+\;k_{m}$ is even, maybe all of these integers are even or exactly two of them are odd. Further, since *Rsum* = − 1, either (2 > *v* ≥ 1 and 1 > *u* ≥ 0) or (1 > *v* ≥ 0 and 2 > *u* ≥ 1). Thus, based on these conditions there are the following eight cases:

- *i* is even, *k_m_* is even, *j_m_* is even, *v* ≥ 1 > *u*. Then, by taking out the integers from the floor function *l* = $\frac{i}{2}-\frac{k_{m}}{2}\;+\;\lfloor \frac{v\;+\;1}{2}\rceil -\frac{j_{m}}{2}\;+\;\lfloor \frac{5\;+\;2u-v}{6}\rceil$. As 2 > *v* ≥ 1, $\left\lfloor \frac{v\;+\;1}{2}\right\rceil$ = 1, and with these conditions, 6 > 5 + 2u−v ≥ 3, which implies $\lfloor \frac{5\;+\;2u-v}{6}\rceil =0$. Therefore, the value of *l* = $\frac{i}{2}-\frac{k_{m}}{2}-\frac{j_{m}}{2}\;+\;1$. However, from $i\;+\;j_{m}\;+\;k_{m}=2$ it is clear that $i=2-j_{m}-k_{m}$, and thus, $\frac{i}{2}=1-\frac{k_{m}}{2}-\frac{j_{m}}{2}$. Consequently, in this case, *l* = *i*.
- *i* is even, *k_m_* is even, *j_m_* is even, *u* ≥ 1 > *v*. *l* = $\frac{i}{2}-\frac{k_{m}}{2}\;+\;\lfloor \frac{v\;+\;1}{2}\rceil -\frac{j_{m}}{2}\;+\;\lfloor \frac{5\;+\;2u-v}{6}\rceil$, however, in this case, $\left\lfloor \frac{v\;+\;1}{2}\right\rceil$ = 0. Further, 9 > 5 + 2u−v ≥ 6, which implies $\lfloor \frac{5\;+\;2u-v}{6}\rceil =1$. Thus, *l* = $\frac{i}{2}-\frac{k_{m}}{2}-\frac{j_{m}}{2}\;+\;1$ = *i*, as in the previous case.
- *i* is even, *k_m_* is odd, *j_m_* is odd and *v* ≥ 1 > *u*. Then, *l* = $\frac{i}{2}-\frac{k_{m}-1}{2}\;+\;\lfloor \frac{v}{2}\rceil -\frac{j_{m}-1}{2}\;+\;\lfloor \frac{2\;+\;2u-v}{6}\rceil$. In this case $\lfloor \frac{v}{2}\rceil =0$ and $\lfloor \frac{2\;+\;2u-v}{6}\rceil =0$, and thus *l* = $\frac{i}{2}-\frac{k_{m}-1}{2}-\frac{j_{m}-1}{2}=\frac{i}{2}-\frac{k_{m}}{2}-\frac{j_{m}}{2}\;+\;1=i$ again.
- *i* is even, *k*_m_ is odd, *j*_m_ is odd and *u* ≥ 1 > *v*. Then, *l* = $\frac{i}{2}-\frac{k_{m}}{2}\;+\;\frac{1}{2}\;+\;\lfloor \frac{v}{2}\rceil -\frac{j_{m}}{2}\;+\;\frac{1}{2}\;+\;\lfloor \frac{2\;+\;2u-v}{6}\rceil$. Again, $\lfloor \frac{v}{2}\rceil =0$ and $\lfloor \frac{2\;+\;2u-v}{6}\rceil =0$, and thus *l* $=\frac{i}{2}-\frac{k_{m}}{2}-\frac{j_{m}}{2}\;+\;1=i$.
- *i* is odd, *k_m_* is even, *j_m_* is odd, *v* ≥ 1 > *u*. Then, by taking out the integers from the floor function, we get *l* = $\frac{i\;+\;1}{2}-\frac{k_{m}}{2}\;+\;\lfloor \frac{v}{2}\rceil -\frac{j_{m}}{2}\;+\;\frac{1}{2}\;+\;\lfloor \frac{2\;+\;2u-v}{6}\rceil$. In this case, we have $\lfloor \frac{v}{2}\rceil =0$ and $\lfloor \frac{2\;+\;2u-v}{6}\rceil =0$, and thus, *l* $=\frac{i}{2}-\frac{k_{m}}{2}-\frac{j_{m}}{2}\;+\;1=i$.
- *i* is odd, *k_m_* is even, *j_m_* is odd, *u* ≥ 1 > *v*. Again, *l* = $\frac{i\;+\;1}{2}-\frac{k_{m}}{2}\;+\;\lfloor \frac{v}{2}\rceil -\frac{j_{m}}{2}\;+\;\frac{1}{2}\;+\;\lfloor \frac{2\;+\;2u-v}{6}\rceil$, where both $\lfloor \frac{v}{2}\rceil =0$ and $\lfloor \frac{2\;+\;2u-v}{6}\rceil =0$, thus *l* = *i*.
- *i* is odd, *k_m_* is odd, *j_m_* is even, *v* ≥ 1 > *u*. *l* = $\frac{i-k_{m}}{2}\;+\;\lfloor \frac{v\;+\;1}{2}\rceil -\frac{j_{m}}{2}\;+\;\lfloor \frac{5\;+\;2u-v}{6}\rceil$, in this case $\lfloor \frac{v\;+\;1}{2}\rceil =1$, but 6 > 5 + 2u−v ≥ 3, which implies $\lfloor \frac{5\;+\;2u-v}{6}\rceil =0$, thus *l* = $\frac{i}{2}-\frac{k_{m}}{2}\;+\;1-\frac{j_{m}}{2}$, that is *l* = *i*.
- *i* is odd, *k_m_* is odd, *j_m_* is even, *u* ≥ 1 > *v*. *l* = $\frac{i-k_{m}}{2}\;+\;\lfloor \frac{v\;+\;1}{2}\rceil -\frac{j_{m}}{2}\;+\;\lfloor \frac{5\;+\;2u-v}{6}\rceil$, but now, $\lfloor \frac{v\;+\;1}{2}\rceil =0$ and 9 > 5 + 2u−v ≥ 6 implies $\lfloor \frac{5\;+\;2u-v}{6}\rceil =1$. Thus, *l* = $\frac{i}{2}-\frac{k_{m}}{2}-\frac{j_{m}}{2}\;+\;1$ = *i*, as in the previous cases.

Thus, in each of the cases *l* = *i*. Let us compute now n by Table 1: n = $i-\frac{2x}{\sqrt{3}}=i-\frac{2}{\sqrt{3}}-\frac{\sqrt{3}\left(i-k\right)}{2}=i-\left(i-k\right)=k$. Further, by computing the last element *m* of the triplet, $m=\frac{i\;+\;k}{2}-y$, and by substituting the *y* coordinate of *r*’, we get $m=\frac{i\;+\;k}{2}-\frac{i-2j\;+\;k}{2}=j$. As, we got that the procedure results in the sum vector *r* = (*i*_3_, *j*_3_, *k*_3_) = (*i*, *j*, *k*) = (*l*, *m*, *n*), that is a valid vector of Ω, the case is proven.

B. This case consists the cases where either *Rsum* = 0 and {$\bar{j}$} ≤ {$\bar{k}$} or *Rsum* = −2 and {$\bar{j}$} ≥ {$\bar{k}$}. The two subcases are analogous, we shell show only the latter one here. The condition *Rsum* = −2 implies that both *u* = *u*_1_ + *u*_2_ > 1 and *v* = *v*_1_ + *v*_2_ > 1. The condition {$\bar{j}$} ≥ {$\bar{k}$} is the same as {*j*} ≥ {k}, and in fact, since these values are negative, equivalent to *u*_1_ + *u*_2_ ≤ *v*_1_ + *v*_2_., i.e., *u* ≤ *v*. The procedure produces a Type B vector *r* = (*i* + *u*_1_ + *u*_2_, *j* + *u*_1_ + *u*_2_, *k* + *u*_1_ + *u*_2_). Remembering the notation *j_m_* = $j_{m1}\;+\;j_{m2}$ and k_m_ = $k_{m1}\;+\;k_{m2}$ and knowing that *j* = *j_m_* − *u*_1_ − *u*_2_, this can be written as *r* = (*i* + *u*_1_ + *u*_2_, *j_m_*, *k* + *u*_1_ + *u*_2_) = (*i* + *u*, *j_m_*, *k* + *u*) = (*i* + *u*, *j_m_*, *k_m_* − *v* + *u*) with the integer coordinate *j_m_*. Let us check the sum of these coordinates: *i* + *u* + *j_m_* + *k_m_* − *v* + *u* = *i* + *j_m_* + *k_m_* + 2*u* − *v* = 2 + 2*u* − *v* (using *i* + *j_m_* + *k_m_* = 2). In the studied case *Rsum* = −2 yielding that *u*, *v* > 1, thus it is larger than 2, we need to apply Step 3b of our procedure: by subtracting 1 from each coordinate, the procedure gives the resulted vector (*i* + *u* − 1, *j_m_* − 1, *k* + *u* −1). Now, let us check if it is correct by computing the Cartesian coordinate pair corresponding to this triplet. $x=\frac{\sqrt{3}\left(i\;+\;u_{1}\;+\;u_{2}-1-k-u_{1}-u_{2}\;+\;1\right)}{2}=\frac{\sqrt{3}\left(i-k\right)}{2}$ and $y=\frac{i\;+\;u_{1}\;+\;u_{2}-1-2j-2u_{1}-2u_{2}\;+\;2\;+\;k\;+\;u_{1}\;+\;u_{2}-1}{2}$ = $\frac{i-2j\;+\;k}{2}$. Thus, exactly the vector *r*’= $\left(\frac{\sqrt{3}\left(i-k\right)}{2},\frac{i-2j\;+\;k}{2}\right)$ is obtained, the sum is correctly computed by our procedure. Now we are checking if the resulted triplet of *r* is a valid vector in Ω, by transforming *r*’ to coordinate triplet, let us say, (*l*, *m*, *n*) in Ω. In Area B, first coordinate *m* is computed (by Table 1), then *l* and *n* are determined. $m=\;\langle \frac{-2y}{3}\rangle$, by substituting *y* we have $m=\;\langle \frac{-2}{3}\cdot \frac{i-2j\;+\;k}{2}\rangle =\;\langle \frac{2j-i-k}{3}\rangle =\langle \frac{3j}{3}-\frac{i\;+\;j\;+\;k}{3}\rangle$. Now, using the facts that $j=j_{m}-u$ and $k=k_{m}-v$, we get the formula $m=\langle j_{m}-\frac{i\;+\;j_{m}\;+\;k_{m}\;+\;2u-v}{3}\rangle$. Applying that $i\;+\;j_{m}\;+\;k_{m}=2$, $m=\langle j_{m}-\frac{2\;+\;2u-v}{3}\rangle =$ $\langle j_{m}-\frac{2}{3}\;+\;\frac{v-2u}{3}\rangle =\langle j_{m}\;+\;\frac{v-2u-2}{3}\rangle$ is obtained. In this case, however, 1 < *u* ≤ *v*, but none of them is larger than 2, which implies that −2 < *v* − 2*u* < 0, thus $-\frac{4}{3}<\frac{v-2u-2}{3}<-\frac{2}{3}$ and this by the rounding operator, clearly implies that $m=j_{m}-1$, which is exactly the same as the value that we have obtained by our procedure. Now, continuing with *l* = $\frac{x\sqrt{3}}{3}\;+\;y\;+\;m=\frac{\sqrt{3}\left(i-k\right)}{2}\cdot \frac{\sqrt{3}}{3}\;+\;\frac{i-2j\;+\;k}{2}\;+\;j_{m}-1=\frac{\left(i-k\right)}{2}\;+\;\frac{i-2j\;+\;k}{2}\;+\;j\;+\;u-1=i\;+\;u-1$, the same value is obtained as the one our procedure gave. Further, we compute $=l-\frac{2x}{\sqrt{3}}=i\;+\;u-1-\frac{2}{\sqrt{3}}\cdot \frac{\sqrt{3}\left(i-k\right)}{2}=$ i + u−1−(i−k)=k + u−1, which is also matching to the result of our procedure by proving this subcase: *r* = (*i*_3_, *j*_3_, *k*_3_) = (*l*, *m*, *n*) = (*i* + *u* − 1, *j* + *u* − 1, *k* + *u* − 1). (The other subcase with the conditions *Rsum* = 0 and {$\bar{j}$} ≤ {$\bar{k}$} can be proven in a similar way.)
C. Finally, the last case consists of the cases in which either *Rsum* = 0 and {$\bar{j}$} > {$\bar{k}$} or *Rsum* = −2 and {$\bar{j}$} < {$\bar{k}$}. In this case, our procedure gives a vector of Type C in an analogous way as we have discussed the previous cases.

The other cases, which are analogous to the presented ones can be proven in a similar way, the technical details left for the reader. □
